# Effects of deep learning on radiologists’ and radiology residents’ performance in identifying esophageal cancer on CT

**DOI:** 10.1259/bjr.20220685

**Published:** 2023-04-22

**Authors:** Koichiro Yasaka, Sosuke Hatano, Masumi Mizuki, Naomasa Okimoto, Takatoshi Kubo, Eisuke Shibata, Takeyuki Watadani, Osamu Abe

**Affiliations:** 1 Department of Radiology, The University of Tokyo Hospital, Tokyo, Japan

## Abstract

**Objective:**

To investigate the effectiveness of a deep learning model in helping radiologists or radiology residents detect esophageal cancer on contrast-enhanced CT images.

**Methods:**

This retrospective study included 250 and 25 patients with and without esophageal cancer, respectively, who underwent contrast-enhanced CT between December 2014 and May 2021 (mean age, 67.9 ± 10.3 years; 233 men). A deep learning model was developed using data from 200 and 25 patients with esophageal cancer as training and validation data sets, respectively. The model was then applied to the test data set, consisting of additional 25 and 25 patients with and without esophageal cancer, respectively. Four readers (one radiologist and three radiology residents) independently registered the likelihood of malignant lesions using a 3-point scale in the test data set. After the scorings were completed, the readers were allowed to reference to the deep learning model results and modify their scores, when necessary.

**Results:**

The area under the curve (AUC) of the deep learning model was 0.95 and 0.98 in the image- and patient-based analyses, respectively. By referencing to the deep learning model results, the AUCs for the readers were improved from 0.96/0.93/0.96/0.93 to 0.97/0.95/0.99/0.96 (*p* = 0.100/0.006/<0.001/<0.001, DeLong’s test) in the image-based analysis, with statistically significant differences noted for the three less-experienced readers. Furthermore, the AUCs for the readers tended to improve from 0.98/0.96/0.98/0.94 to 1.00/1.00/1.00/1.00 (*p* = 0.317/0.149/0.317/0.073, DeLong’s test) in the patient-based analysis.

**Conclusion:**

The deep learning model mainly helped less-experienced readers improve their performance in detecting esophageal cancer on contrast-enhanced CT.

**Advances in knowledge:**

A deep learning model could mainly help less-experienced readers to detect esophageal cancer by improving their diagnostic confidence and diagnostic performance.

## Introduction

It is a well-established fact that esophageal cancer is associated with poor prognosis.^1^ According to the American Cancer Society, the 5 year relative survival rate of localized, regional, and distant stages for this type of cancer is reported to be 47%, 25%, and 5%, respectively.^2^ Screening may be helpful in terms of detecting esophageal cancer at the earlier stages of the disease. However, because no screening test has been shown to reduce the risk of death from this type of cancer, screening the general public for esophageal cancer is currently not recommended in the United States.^2^

Esophageal cancer is relatively common among all the cancers. According to the World Health Organization, the number of new esophageal cases in 2020 was the eighth highest among all the cancers.^3^ Cigarette smoking is known to be associated with the incidence of esophageal cancer.^1^ Because smoking is also a risk factor for the development of other types of cancer, such as head and neck cancer,^4^ lung cancer,^5^ pancreatic cancer,^6^ and abdominal aortic aneurysms,^7^ it is possible the esophageal cancer could be depicted incidentally in CT images performed for other diagnostic purposes. This is also especially true because of the rapidly increasing use of CT in clinical settings.^8^

Recently, applications of deep learning with convolutional neural network to radiological imaging diagnosis is gaining wide attention.^9,10^ It has been reportedly possible for deep learning models to detect,^11,12^ stage,^13,14^ and classify^15,16^ lesions from radiological images. Developing a deep learning model that can effectively detect esophageal cancer on CT images before symptomatic may have a practical value improving patient prognosis and treatment outcomes.

More recently, how to deploy deep learning to clinical settings is also gaining attention. In this context, the role of deep learning algorithms is beginning to be regarded as supporting and helping radiologists’ and radiology residents’ decision-making processes rather than replacing them.^17^ Consequently, it is essential to assess whether deep learning models can help radiologists and radiology residents to detect incidental esophageal cancer. Therefore, the purpose of this study was to investigate whether a deep learning model can support radiologists and radiology residents in detecting esophageal cancer on contrast-enhanced CT images.

## Methods and materials.

This retrospective, single-center study was approved by our institutional review board. The requirement for obtaining written informed consent was waived.

### Overview

**Figure 1.**
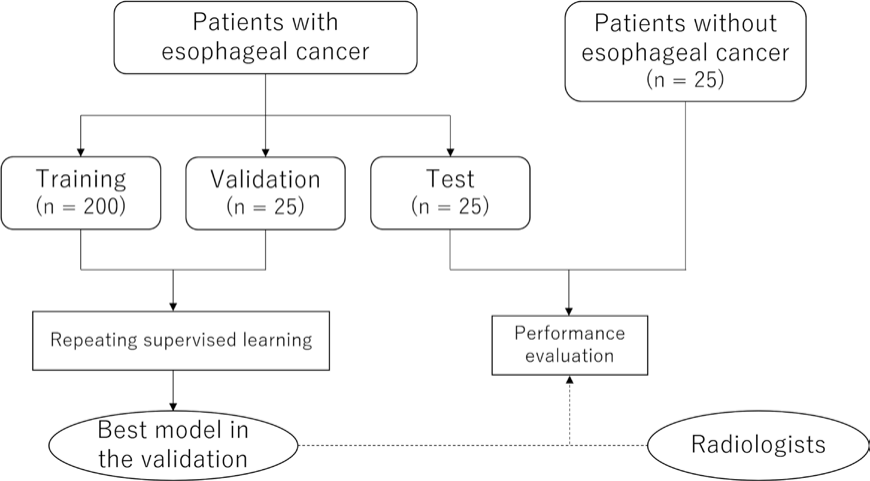
Flowchart of the study.

Figure 1 illustrates the overview of this study. First, we developed a deep learning model that could determine whether esophageal cancer is depicted in a single CT image. We performed supervised learning by using a training data set, and the model performance was assessed with a validation data set. Consequently, to avoid overestimating the model’s performance due to the optimization of hyperparameters, the performance of the best model in the validation data set was further tested with the test data set, which comprised patients with and without esophageal cancer. Patients without esophageal cancer were included in order to analyze the detection performance in a patient-based analysis. Finally, radiologist’s and radiology residents’ performance in detecting esophageal cancer with and without reference to the deep learning model’s results was evaluated and compared.

### Patients

We searched for consecutive patients with esophageal cancer by using the picture archiving and communication system. Inclusion criteria were as follows: patients who underwent contrast-enhanced CT, including chest, before treatment; patients with visible esophageal cancer on CT; patients who underwent upper gastrointestinal tract endoscopy; and patients with a histopathological diagnosis of esophageal cancer. For the test data set, age- and gender-matched patients without esophageal cancer who underwent contrast-enhanced CT, including chest, were also selected and were included.

### Input data: CT imaging and preprocessing

Contrast-enhanced CT examinations were performed with scanners from two vendors (Aqulion ONE and Aquilion PRIME from Canon Medical Systems [Tochigi, Japan] and Discovery CT 750HD and Revolution CT from GE Healthcare [WI]). Pertaining to the tube current, the automatic exposure control technique was used, the standard deviation was set to 13.0 for Canon-CT, and the noise index was set to 11.36 for GE-CT. Other scan and reconstruction parameters were as follows: tube voltage, 120 kVp; reconstruction algorithm, filtered backprojection; reconstruction plane, axial; and slice thickness/interval, 5/5 mm. The concentration and total volume of contrast enhancement material was determined based on the patients’ body weight (350 and 370 mg of iodine per milliliter for patients who weighed <60 and >60 kg, respectively; the total volume (ml) was determined by multiplying the body weight (in kilograms) by 2, with an upper limit of 100 ml). Scans were performed 90 s after starting injection.

CT images were further preprocessed with the Python 3.6.4 programming language (https://www.python.org/), Pillow 5.0.0 (https://pillow.readthedocs.io/en/stable/#), and pydicom 1.2.2 (https://pydicom.github.io/pydicom/stable/#). To facilitate the deep learning process, the lower middle part (with 256 × 256 pixels and fixed crop location) was cropped from the original CT image with 512 × 512 pixels and was used as input data. This means that parts of the images which did not include esophagus were omitted. This process was performed fully automatically. The crop location was determined empirically by referencing some CT images from the training data. With respect to the training data set, augmentation was performed by generating images with slightly altered cropping locations, rotated images, flipped images, and noise added images. Data augmentation was also performed with the Python 3.6.4, Pillow 5.0.0, and pydicom 1.2.2 packages. With augmentation, the data set was expanded 32- and 7-fold for CT images with and without esophageal cancer, respectively (different numbers were adopted so as to balance the number of positive and negative images).

### Reference standard: the presence of esophageal cancer

By referencing to the upper gastrointestinal endoscopy report and histopathological report, whether esophageal cancer is depicted in a CT image (1 = esophageal cancer present and 0 = esophageal cancer absent) was determined. With respect to the training and validation data sets, a radiologist (Radiologist #1, with diagnostic imaging experience of 11 years after 2 years of post-graduate training as intern) made the reference standard. As for the test data set, because we thought that the deep learning model’s performance in this data set would be clinically more relevant, another radiologist (Radiologist #2, with diagnostic imaging experience of 8 years after 2 years of post-graduate training as intern) was also involved in establishing the reference standard, and evaluation was performed in a consensus reading by the two radiologists. All the labelling process was performed before the supervised training of the convolutional neural network.

### Supervised learning of convolutional neural network

Deep learning was performed by the Radiologist #1 with a computer equipped with 128 GB RAM, a Core i9-7900X central processing unit (Intel) and a Quadro P6000 graphics processing unit (NVIDIA). The programming language of Python 3.6.4 and a deep learning framework of Chainer 4.0.0 (https://chainer.org/) were used.

**Figure 2.**
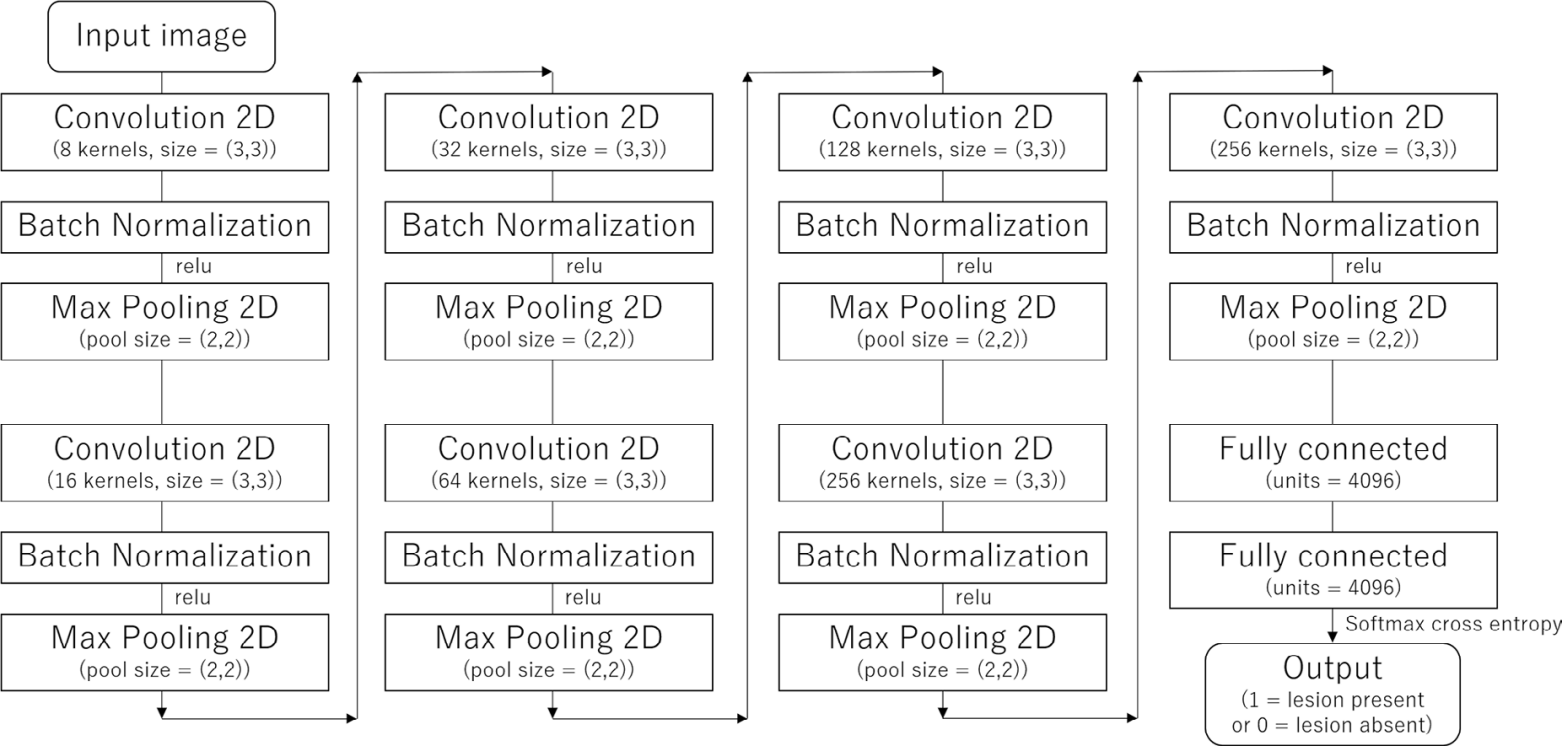
The structure of the convolutional neural network used in this study. 2*D*, two dimensional; relu, rectified linear unit.

Based on the date of the CT examination, patient data were split into training (*n* = 200 [from December 2014 to November 2019]), validation (*n* = 25 [from November 2019 to August 2020]), and test (*n* = 25 [from August 2020 to May 2021]) data sets. In the supervised learning of convolutional neural network, a cropped CT image (please refer to ***Input data: CT imaging and preprocessing*** subsection) and the binary data for the presence of esophageal cancer (please refer to ***Reference standard: the presence of esophageal cancer*** subsection) were used as input and teaching data, respectively. Hyperparameters were empirically optimized with the use of training and validation data (*i.e.* test data were not used in optimizing hyperparmeters). The structure of the convolutional neural network used in this study is illustrated in Figure 2. Additional hyperparameters used in this study were the following: the number of epoch, 20; minibatch size, 15; and the optimizer, Adam. Training and validation were performed 15 times for each session while incrementally increasing the number of patients for the training group by 10 patients (Session 1 [10 patients for the training], Session 2 [20 patients for the training], …, and Session 20 [200 patients for the training]). This resulted in the generation of 15 models for each session. The average performance among the 15 models in each session was recorded. The model that showed the best diagnostic performance among the 15 models in Session 20 was selected, and this model was applied to the test data set.

### Lesion detection by readers

One radiologist (Reader A with diagnostic imaging experience of 11 years after 2 years of post-graduate training as intern) and three radiology residents (Readers B, C, and D with diagnostic imaging experience of 3 years, 1 year, and 3 months, respectively after 2 years of post-graduate training as intern) were involved in the lesion detection tests. The four readers were blinded to the clinical and histopathological information, and they were also not informed of the purpose of this study. They were asked to independently detect lesions with malignant potential on the CT images using a 3-point scale (diagnostic score; 3 = lesion present, 2 = not sure for the presence of lesion, and 1 = lesion absent). The 50 individual data sets were presented in a randomized sequence.

After the lesion detection test was performed for all the patient, the four readers were allowed to reference the output scores for each CT slice derived from the deep learning model and to modify the above-mentioned score, when necessary. The readers were also informed of the deep learning model performance and the cut-off value that achieved the Youden index in the validation data set (not in the test data set). Furthermore, they were also asked to evaluate whether the deep learning model was helpful in improving the diagnostic confidence of detecting esophageal cancer with a 5-point scale (usefulness of the deep learning model; 2 = very helpful, 1 = helpful, 0 = undetermined, −1 = not helpful, and −2 = not helpful at all).

### Statistics

Statistical analyses were performed with R v. 2.4.0 (https://www.r-project.org/). Continuous variables were expressed as mean and standard deviation and were compared by using Student’s *t*-tests. In contrast, binary variables were compared by using the χ^2^ test. To evaluate the diagnostic performance in detecting esophageal cancer, receiver operating characteristic curve analysis was performed, and the area under the receiver operating characteristic curve (AUC) was calculated. Image-based analysis was performed in the validation group, whereas image-based and patient-based analyses were performed in the test group. The highest score among all CT slices from one patient was registered in patient-based analysis. To compare the readers’ performance with and without referencing to the results of the deep learning model, the DeLong’s test for AUC and the McNemar’s test for accuracy, sensitivity, and specificity were performed. Comparisons between diagnostic scores with and without referencing to the results of the deep learning model were then performed by using paired *t*-tests.

## Results

### Patients

In this study, 200, 25, and 25 consecutive patients with esophageal cancer were included in the training, validation, and test groups, respectively (Figure 1). In addition, 25 age- and gender-matched patients without esophageal cancer were selected as negative controls in the test group. The total number of CT slices for the training, validation, and test groups were 11429, 1418, and 3133, respectively. Table 1 summarizes the background information of the 250 patients and 25 controls.

**Table 1.** Patient background information

	Training	Validation	Test (esophageal cancer positive)	Test (esophageal cancer negative)	Comparison
Age (years)	67.4 ± 9.9	68.4 ± 11.8	71.3 ± 9.8	67.2 ± 11.9	*p* = 0.193
Male/Female	169/31	23/2	20/5	21/4	*p* = 1.000
Biopsy/Surgery	75/125	11/14	9/16	N/A	N/A
Main location					N/A
Cervical	15	3	0	N/A	
Upper thoracic	36	6	7	N/A	
Middle thoracic	88	11	12	N/A	
Lower thoracic	61	5	6	N/A	
Length of esophageal cancer (mm)	54.6 ± 28.2	46.6 ± 22.8	41.2 ± 23.9	N/A	N/A
T stage					*p* = 0.652
T1	27	4	3	N/A	
T2	72	8	14	N/A	
T3	81	11	7	N/A	
T4	20	2	1	N/A	
Pathology					N/A
SCC	178	24	24	N/A	
adenocarcinoma	13	0	0	N/A	
Other	9	1	1	N/A	

N/A, not applicable; SCC, squamous cell carcinoma.

For continuous parameters, comparisons between esophageal cancer positive and negative tests were performed by using the Student’s *t*-test, whereas the chi-square test was used for categorical parameters.

### Relationship between the number of patients and deep learning model’s performance

**Figure 3.**
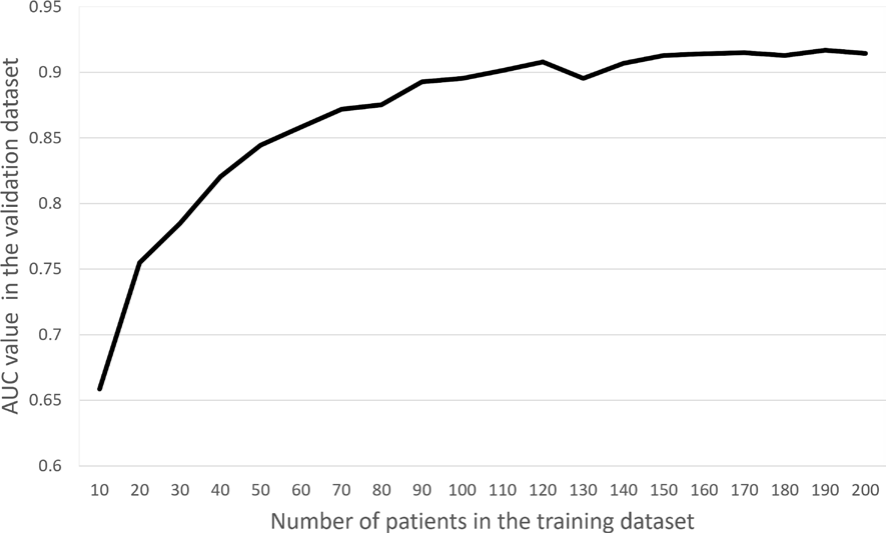
Relationship between the number of patients in the training data set and the diagnostic performance in the validation data set. AUC, area under the receiver operating characteristic curve.

The relation between the number of patients used in the training data set and the performance of the CNN in the validation data set is shown in Figure 3. As the number of patients in the training data set increased, CNN performance tended to improve. This trend became less obvious when the number of patients in the training data set exceeded 150.

### The performance of the trained model in the test data set

In the validation data set, the AUC of the best model was 0.92, and the accuracy/sensitivity/specificity was 0.86/0.85/0.87, respectively, by using the threshold value of 0.189 that achieved the Youden index. By applying the best deep learning model to the test data set, the AUC was calculated as 0.95 and 0.98 in the image- and patient-based analyses, respectively. By applying the cut-off value that achieved the Youden index, the accuracy/sensitivity/specificity in the test group were 0.92/0.87/0.92 and 0.96/0.96/0.96 in the image- and patient-based analyses, respectively (Table 2).

**Table 2.** The performance of the DL model and readers in the test data set

		AUC	Accuracy	Sensitivity	Specificity
Image-based analysis					
DL model		0.95	0.92 (2876/3133)	0.87 (213/245)	0.92 (2663/2888)
Reader A	Without DL model	0.96	0.96 (3001/3133)	0.97 (238/245)	0.96 (2763/2888)
	With DL model	0.97	0.96 (3012/3133)	0.98 (239/245)	0.96 (2773/2888)
	Comparison	0.100	0.091	1.000	0.123
Reader B	Without DL model	0.93	0.98 (3065/3133)	0.88	0.99 (2850/2888)
	With DL model	0.95	0.98 (3079/3133)	0.90 (220/245)	0.99 (2859/2888)
	Comparison	0.006^a^	0.001^a^	0.074	0.015^a^
Reader C	Without DL model	0.96	0.94	0.95 (233/245)	0.94 (2723/2888)
	With DL model	0.99	0.95 (2971/3133)	1.00 (245/245)	0.94 (2726/2888)
	Comparison	<0.001^a^	0.077	0.002^a^	0.877
Reader D	Without DL model	0.93	0.96 (3016/3133)	0.90 (220/245)	0.97 (2796/2888)
	With DL model	0.96	0.95 (2973/3133)	0.96 (235/245)	0.95 (2738/2888)
	Comparison	<0.001^a^	<0.001^a^	0.001^a^	<0.001^a^
Patient-based analysis					
DL model		0.98	0.96 (48/50)	0.96 (24/25)	0.96 (24/25)
Reader A	Without DL model	0.98	0.98 (49/50)	1.00 (25/25)	0.96 (24/25)
	With DL model	1.00	1.00 (50/50)	1.00 (25/25)	1.00 (25/25)
	Comparison	0.317	1.000	N/A	1.000
Reader B	Without DL model	0.96	0.96 (48/50)	0.92 (23/25)	1.00 (25/25)
	With DL model	1.00	1.00 (50/50)	1.00 (25/25)	1.00 (25/25)
	Comparison	0.149	0.480	0.480	N/A
Reader C	Without DL model	0.98	0.98 (49/50)	0.96 (24/25)	1.00 (25/25)
	With DL model	1.00	1.00 (50/50)	1.00 (25/25)	1.00 (25/25)
	Comparison	0.317	1.000	1.000	N/A
Reader D	Without DL model	0.94	0.94 (47/50)	0.88 (22/25)	1.00 (25/25)
	With DL model	1.00	0.98 (49/50)	0.96 (24/25)	1.00 (25/25)
	Comparison	0.073	0.480	0.480	N/A

AUC, area under the curve; DL, deep learning; N/A, not applicable.

Accuracy, sensitivity, and specificity were calculated by using the threshold that achieved the Youden index. Comparisons between AUC values were performed with the DeLong’s test, and comparisons among accuracy, sensitivity, and specificity were performed by using the McNemar’s test.

^a^statistically significant difference.

### Readers’ performance with and without referencing to the results of the deep learning model

**Figure 4.**
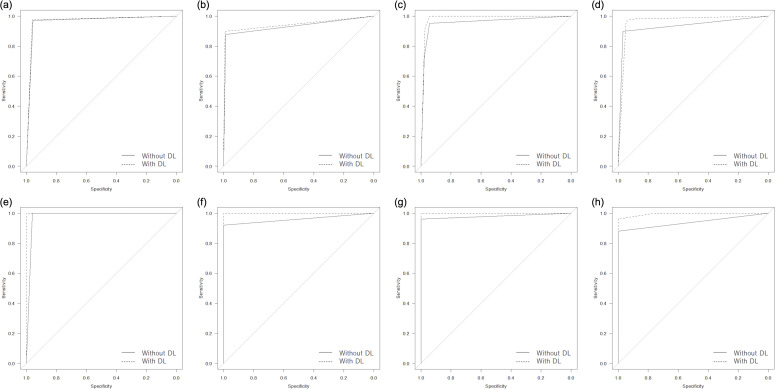
Receiver operating characteristic curves in detecting esophageal cancer for Readers A (**a, e**), B (**b, f**), C (**c, g**), and D (**d, h**) for image-based (a, b, c, and d) and patient-based (e, f, g, and h) analyses. DL, deep learning.

**Figure 5.**
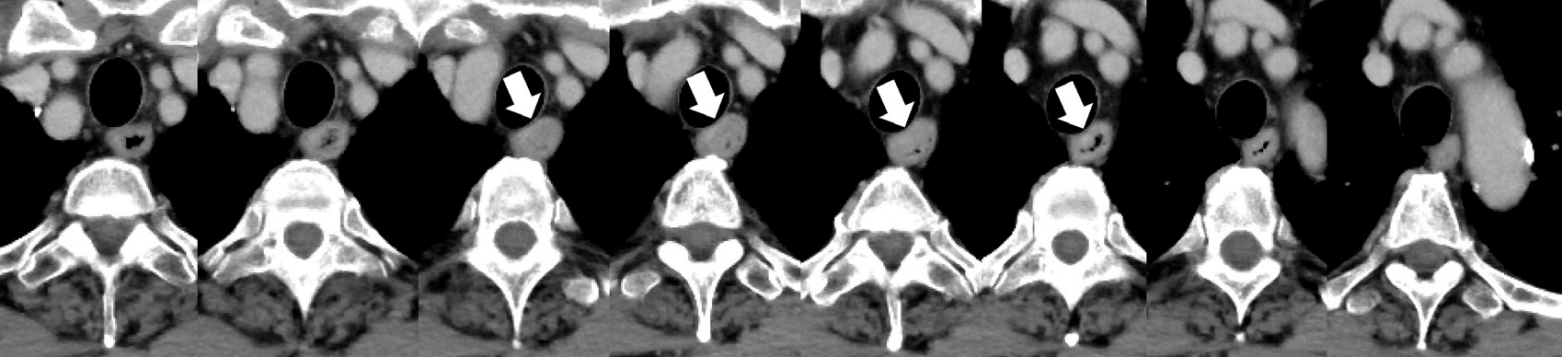
Contrast-enhanced axial CT images (left to right: cranial to caudal) of a 71-year-old male patient in the test group with esophageal cancer (indicated by white arrows) located to the upper part of the esophagus. The output score of the deep learning model for this patient was 0.997 in the patient-based analysis, which was above the threshold value of 0.189 determined by using the validation data set, and was diagnosed as positive. In the patient-based analysis, the four readers scored 3 (lesion present)/1 (lesion absent)/2 (not sure for the presence of lesion)/1 without referencing to the results of the deep learning model. However, when referencing to the results of our model, scores were improved to 3/3/2/2.

**Figure 6.**
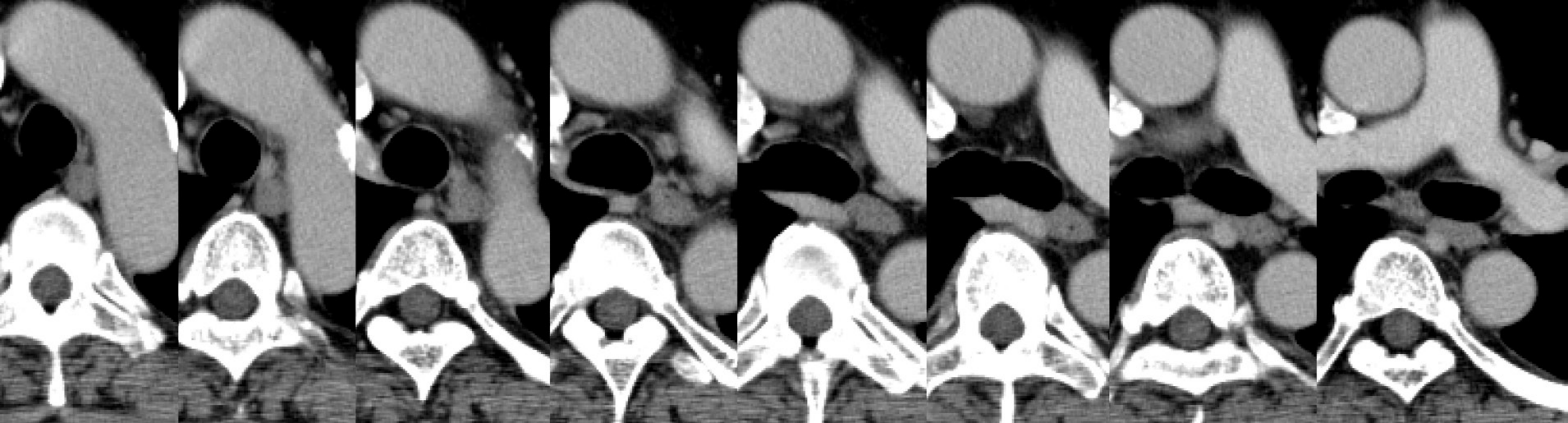
Contrast-enhanced axial CT images (left to right: cranial to caudal) of a 70-year-old male patient in the test group without esophageal cancer. The output scores of the deep learning model in the image-based analysis were 0/0.19/0.93/0.58/0.12/0.03/0.01/0 from left to right. Reader C rated these images as 1 (lesion absent)/1/1/1/1/1/1/1 and 1/1/2 (not sure for the presence of lesion)/2/1/1/1/1 from left to right without and with referencing to the results of the deep learning model, respectively.

In the image-based analysis (Table 2), referencing to the results of the deep learning model improved AUCs in all readers from 0.96/0.93/0.96/0.93 to 0.97/0.95/0.99/0.96, and statistically significant differences were noted for radiology residents (*i.e.* Readers B, C, and D) (*p* < 0.006) (Figure 4). The sensitivity of less-experienced readers showed a tendency for improvement from 0.88/0.95/0.90 to 0.90/1.00/0.96 (Figure 5), and statistically significant differences were observed for Readers C and D (*p* < 0.002). However, the specificity of Reader D deteriorated from 0.97 to 0.95 when referencing to the results of the deep learning model (*p* < 0.001) (Figure 6). These results were also reflected in the diagnostic confidence scores, which are described in Table 3.

**Table 3.** Diagnostic score and usefulness of the deep learning model in the test data set

	Diagnostic score			Usefulness of the DL model (95% confidence interval)
	Without the DL model	With the DL model	Comparison	
Esophageal cancer positive				
Image-based analysis				
Reader A	2.94 ± 0.33	2.95 ± 0.31	0.318	N/A
Reader B	2.76 ± 0.66	2.79 ± 0.61	0.032^a^	N/A
Reader C	2.68 ± 0.56	2.91 ± 0.28	<0.001^a^	N/A
Reader D	2.71 ± 0.64	2.94 ± 0.31	<0.001^a^	N/A
Patient-based analysis				
Reader A	3.00 ± 0.00	3.00 ± 0.00	N/A	0.60 ± 0.82 (0.26–0.94)
Reader B	2.84 ± 0.55	2.96 ± 0.20	0.185	0.36 ± 0.64 (0.10–0.62)
Reader C	2.64 ± 0.57	2.88 ± 0.33	0.011^a^	1.60 ± 0.58 (1.36–1.84)
Reader D	2.64 ± 0.70	2.96 ± 0.20	0.018^a^	1.16 ± 0.37 (1.01–1.31)
Esophageal cancer negative				
Image-based analysis				
Reader A	1.09 ± 0.41	1.08 ± 0.40	0.243	N/A
Reader B	1.03 ± 0.23	1.02 ± 0.19	0.002^a^	N/A
Reader C	1.08 ± 0.34	1.08 ± 0.36	0.371	N/A
Reader D	1.06 ± 0.34	1.13 ± 0.46	<0.001^a^	N/A
Patient-based analysis				
Reader A	1.08 ± 0.40	1.00 ± 0.00	0.327	−0.56 ± 0.96 (−0.96 to −0.16)
Reader B	1.00 ± 0.00	1.00 ± 0.00	N/A	0.16 ± 0.62 (−0.10 to 0.42)
Reader C	1.00 ± 0.00	1.00 ± 0.00	N/A	1.44 ± 0.51 (1.23–1.65)
Reader D	1.00 ± 0.00	1.24 ± 0.44	0.011^a^	0.52 ± 0.65 (0.25–0.79)

DL, deep learning; N/A, not applicable.

Comparisons with and without the deep learning model were performed by using the paired *t*-test.

^a^statistically significant difference.

In the patient-based analysis, both AUC and accuracy of all readers tended to increase by referencing to the results of the deep learning model, with Readers A, B, and C reaching perfect performance (*i.e.* reached 1.00) (Table 2). In positive diagnosis of esophageal cancer, the diagnostic confidence of radiology residents (*i.e.* Readers B/C/D) improved from 2.84/2.64/2.64 to 2.96/2.88/2.96, and statistically significant differences were noted for Readers C and D (*p* < 0.018) (Table 3). However, in negative diagnosis for patients without esophageal cancer, the diagnostic confidence of Reader D deteriorated from 1.00 to 1.24 (*p* = 0.011).

The scores regarding the usefulness of the deep learning model in patients with and without esophageal cancer are summarized in Table 3. Overall, the three less-experienced readers assessed the deep learning model as significantly helpful in detecting esophageal cancer (scores [with 95% confidence intervals] of 0.26 [0.08–0.44], 1.52 [1.37–1.67], and 0.84 [0.66–1.02] by Readers B, C, and D, respectively). However, the score of the radiologist (*i.e.* Reader A), was below the significance level 0.02 [−0.28–0.32].

## Discussion

In this study, we developed a deep learning model that detects esophageal cancer on contrast-enhanced CT images. Our findings showed that this model could exhibit high levels of diagnostic performance. In addition, the model was found to increase the confidence and improve the diagnostic performance of less-experienced readers in terms of detecting esophageal cancer. In fact, the accuracy of readers with imaging experience of more than 1 year reached perfect levels when referencing to the results of the deep learning model.

Overall, the deep learning model achieved high performance in detecting esophageal cancer. A previous research study, which also developed a deep learning model to detect esophageal cancer on CT images,^18^ reported that the AUC/accuracy/sensitivity/specificity of the model were 0.91/0.84/0.72/0.90, respectively. In contrast, our model achieved AUC/accuracy/sensitivity/specificity values of 0.95/0.92/0.87/0.92 and 0.98/0.96/0.96/0.96 in the image- and patient-based analyses, respectively, which were superior to the previously developed model. Furthermore, the previous research study compared the performance of the deep learning model to the performance of the radiologists. However, the fundamental role of deep learning models is to provide support to radiologists and radiology residents rather than replacing them in clinical settings. Therefore, obtaining data how the deep learning model affects, or can affect, the readers’ performance is significantly more necessary compared to collecting data that indicate whether deep learning models can surpass the readers’ performance. Consequently, this study evaluated the radiologist’s and radiology residents’ performance with and without referencing to the results of the deep learning model.

Our findings reveal that when making a positive diagnosis in patients with esophageal cancer, the sensitivity as well as the diagnostic scores of less-experienced readers showed a tendency for improvement. More specifically, among 25 patients, the diagnosis of two, one, and two patients was corrected as positive for esophageal cancer by Readers B, C, and D, respectively, after referencing to the results of the deep learning model. Consequently, the application of this deep learning model in actual clinical settings may allow the diagnosis of esophageal cancer on contrast-enhanced CT images performed for other purposes.

When diagnosing patients without esophageal cancer as negative, the specificity of the experienced radiologist reached perfect levels after referencing to the results of the deep learning model. In contrast, this process deteriorated the diagnostic score of the radiology resident with imaging experience of only a few months. The thickness of esophageal wall thickness varies depending on the degree of distension and there is no clear definition of esophageal wall thickness.^19^ We assume that Reader D was not familiar enough with such imaging findings to correctly dismiss the pseudopositive findings suggested by the deep learning model. However, even in this case, the patients’ scores were rated as 2 (not sure for the presence of lesions) and not as 3 (lesion present) in our implemented 3-point scale.

Furthermore, we also assessed the relationship between the number of patients in the training data set and the performance of the deep learning model in the validation data set. Theoretically, the performance of the deep learning model is thought to be improved as the number of patients in the training data set increases, and our results were consistent with this prediction. In our data set, the model’s performance reached almost a plateau level when the number of patients in the training group was over 150. Therefore, it can be suggested that the majority of the image finding patterns pertaining to esophageal cancer can be covered with these 150 patient images.

There are some limitations in this study. First, the number of patients included in the training data set was relatively small. However, the performance of the deep learning model was found to reach almost a plateau level when the number of patients in the training data set was above 150. Therefore, we assume that even when employing a greater number of patients, the performance would still not increase dramatically. Second, patients with only visible esophageal cancer on CT images were included in this study, since it can be very challenging to prepare a reference standard for the early stages of esophageal cancer. Our model would not be effective in detecting esophageal cancer at very early stages. Third, because some patients underwent chemotherapy before the surgery, pathological proof for esophageal cancer was confirmed only from biopsy specimen for them. Fourth, patients in the esophageal cancer negative group in the test data set did not undergo endoscopy. However, considering the incidence of esophageal cancer (6.3 per 100,000 population),^20^ it would be reasonable to assume that the risk patients with esophageal cancer were misclassified to “patients without esophageal cancer” group would be very low. Fifth, because we cropped lower middle part of CT images in preprocessing, our model would not be applicable to patients scanned with prone or lateral positions. Finally, the performance of our model was not externally validated. Further research is needed with a large number of patients from multicenters in the test group. Lager patient numbers may also provide enough patients for stage by stage comparison, providing data on the effectiveness of the deep learning model in earlier cancer stage.

## Conclusion

In conclusion, the deep learning model to detect esophageal cancer on contrast-enhanced CT was helpful mainly for less-experienced radiology residents in terms of improving their diagnostic performance. However, we should be aware that there might be a risk that radiology residents with limited imaging experience, *e.g.* of just a few months, can become less confident in diagnosing patients without esophageal cancer as negative while referencing to the results of the deep learning model.
